# Pleiotropic effects of *Mentha longifolia* L. extract on the regulation of genes involved in inflammation and apoptosis induced by *Clostridioides difficile* ribotype 001

**DOI:** 10.3389/fmicb.2023.1273094

**Published:** 2023-10-27

**Authors:** Hamideh Raeisi, Masoumeh Azimirad, Elham Abdemohamadi, Raffaele Pezzani, Mohammad Reza Zali, Abbas Yadegar

**Affiliations:** ^1^Foodborne and Waterborne Diseases Research Center, Research Institute for Gastroenterology and Liver Diseases, Shahid Beheshti University of Medical Sciences, Tehran, Iran; ^2^Phytotherapy Lab, Department of Medicine (DIMED), University of Padova, Padua, Italy; ^3^Accademia Italiana di Fitoterapia, Brescia, Italy; ^4^Gastroenterology and Liver Diseases Research Center, Research Institute for Gastroenterology and Liver Diseases, Shahid Beheshti University of Medical Sciences, Tehran, Iran

**Keywords:** *M. longifolia* extract, *Clostridioides difficile*, ribotype 001, Tox-S, inflammation, apoptosis

## Abstract

**Introduction:**

The dramatic increase in multidrug-resistance of *Clostridioides difficile* isolates has led to the search for new complementary medicines against *C. difficile* infection (CDI). In this study, we aimed to examine the inhibitory effects of hydroethanolic extract of *Mentha longifolia* L. (ETOH-ML) on the growth of *C. difficile* RT001 and its toxigenic cell-free supernatant (Tox-S)-induced inflammation and apoptosis.

**Methods:**

The active phytochemical components of ETOH-ML were detected using GC and HPLC. The antimicrobial properties of the extract were examined against *C. difficile* RT001. Furthermore, cell viability and cytotoxicity of Caco-2 and Vero cells treated with various concentrations of ETOH-ML, Tox-S of *C. difficile* RT001, and their combination were assessed. Anti-inflammatory and anti-apoptotic activities of ETOH-ML were explored in Tox-S stimulated Caco-2 cells using RT-qPCR.

**Results:**

Based on our results, rosmarinic acid was the main phytochemical component of ETOH-ML. The extract showed significant antimicrobial activity against *C. difficile* RT001 by agar dilution and broth microdilution methods. Moreover, ETOH-ML at concentrations of <25 μg/ml had no significant effect on cell viability compared to untreated cells. Treatment cells with the extract (10 or 25 μg/ml) significantly increased the cell viability and reduced the percentage of cell rounding in Caco-2 and Vero cells treated by Tox-S, respectively (*P* < 0.0001). Co-treatment of Tox-S stimulated Caco-2 cells with ETOH-ML showed significant anti-inflammatory and anti-apoptotic activities by downregulating the gene expression level of IL-8, IL-1β, TNF-α, iNOS, TGF-β, NF-κB, Bax, and caspase-3, while upregulating the expression level of Bcl-2.

**Discussion:**

Our results demonstrated for the first time the antimicrobial, anti-inflammatory, and anti-apoptotic effects of *M. longifolia* extract on *C. difficile* RT001 and its Tox-S. However, further research is needed to evaluate the potential application of *M. longifolia* extract on CDI treatment in clinical setting.

## Introduction

*Clostridioides difficile* (*C. difficile*) is a Gram-positive, toxin-producing, and spore-forming anaerobic bacterium that is known as the major cause of healthcare-associated infections with significant rates of morbidity and mortality (Kelly et al., 2021). The main symptoms of *C. difficile* infection (CDI) are based on the secretion of two large toxins, toxin A (TcdA) and toxin B (TcdB), that affect intestinal epithelial cells (IECs) by glycosylation and inactivation of Rho/Ras proteins (Shen, 2012). Inactivation of Rho proteins by toxins can lead to downstream cellular changes, including cytoskeleton disintegration, impairment of tight junctions, extensive damage to the large intestine, and in some cases pseudomembranous colitis (PMC) (Di Bella et al., 2016; Czepiel et al., 2019). Additionally, the secretion of these toxins into gastrointestinal (GI) tract triggers intracellular signaling cascades, which induce acute inflammation and cell apoptosis (Shen, 2012). These events can damage the colonic mucosa of patients and cause severe chronic diarrhea (Czepiel et al., 2019). The hypervirulent strains of *C. difficile* can produce a third toxin, known as binary toxin *C. difficile* transferase (CDT), and which can aggravate PMC and cause more severe disease (Di Bella et al., 2016).

Conventional treatment for mild to moderate CDI includes antibiotic therapy, especially fidaxomicin, vancomycin, or metronidazole (Kelly et al., 2021). In recent years, the indiscriminate use of these non-selective antibiotics has been regarded as a major factor in CDI development, which leads to further irritating gut dysbiosis and the emergence of antibiotic-resistant strains of *C. difficile* (Spigaglia, 2016). Additionally, repeated failed antibiotic treatments can cause recurrence of CDI (rCDI) in approximately 15–35% of cases (Song and Kim, 2019). Recently, plant-derived compounds have received widespread attention as pharmacological agents (Dehelean et al., 2021). Plant extracts are complex mixtures of different compounds that are regarded as the most efficient and safe sources of medications and have multiple therapeutic uses in human medicine due to their antimicrobial, antioxidant, and anti-inflammatory properties (Allegra, 2019; Manandhar et al., 2019). These properties of phytochemicals may modulate the immune response triggered by pathogens; thus, these molecules can be considered as potential supplemental therapies (Dehelean et al., 2021). So far, the antimicrobial activity of various active natural components against *C. difficile* has been reported, including garlic, ginger, onion bulb, cinnamon, turmeric, chili pepper, and thymol components (Roshan et al., 2017; Azimirad et al., 2022). Notably, some of these extracts showed protective activity against destructive effects of *C. difficile* toxins, demonstrating that herbal extracts and essential oils could be considered as potential therapeutic candidates for treating CDI (Roshan et al., 2017, 2018).

*Mentha longifolia* L., known as an aromatic plant, is one of the medicinal plants having bioactive properties (Mohammadi and Asadi-Gharneh, 2018). Formulations derived from *M. longifolia* are considered safe and less toxic than traditional medicine, and they can be used for the treatment of several diseases, including nausea, bronchitis, anorexia, ulcerative colitis, and liver maladies (Mikaili et al., 2013; Farzaei et al., 2017). Several *in vitro* and *in vivo* studies have supported various biological features of *M. longifolia* and its derivatives, including antioxidant (Iqbal et al., 2013), anti-inflammatory (Karimian et al., 2013), gastro-protective, antispasmodic, antiemetic, carminative, and analgesic activities (Farzaei et al., 2017). Additionally, the antibacterial effects of *M. longifolia* extracts on several bacterial strains have been reported in the last decades (Elansary et al., 2020).

Among different toxigenic *C. difficile* ribotypes (RTs), RT001 is widely prevalent in many European and Asian countries (Freeman et al., 2015; Davies et al., 2016; Liu et al., 2018; Azimirad et al., 2020a; Collins et al., 2020; Abdrabou et al., 2021). Additionally, since the strains of *C. difficile* RT001 are toxigenic, the toxins produced by these strains may trigger inflammatory responses in the gut, which can cause tissue damages (Shen, 2012). Thus, the use of antimicrobial and anti-inflammatory agents could be a promising and complementary therapeutic option with potential preventive effects on CDI development mainly through modulation of inflammation (Karimian et al., 2013; Eftekhari et al., 2021). This study was undertaken to examine the antimicrobial activities of hydroethanolic extract of *M. longifolia* (ETOH-ML) on a toxigenic *C. difficile* clinical strain (RT001). The inhibitory effects of the extract on cytotoxicity induced by toxigenic cell-free supernatant (Tox-S) of *C. difficile* RT001 were determined using Caco-2 and Vero cells. In addition, the modulatory effects of the extract on expression level of the genes involved in inflammation and apoptosis were assessed in Caco-2 cells stimulated by Tox-S.

## Materials and methods

### Preparation of *M. longifolia* extract

*Mentha longifolia* plant was collected in March 2021 from various regions of Chaharmahal and Bakhtiari Province, Southwestern Iran. The plants (leaves and stems) were rinsed with distilled water, dried, chopped, and used for preparing the hydroethanolic extract. Twenty grams of powdered plant was suspended in 200 ml of ethanol (70% v/v) and macerated in the dark for 24 h at a controlled temperature, and under continuous mechanical stirring. The extract was filtered through a paper filter and centrifuged at 4000 × *g* for 15 min. The supernatant was filtered through 0.22 μm pore filters (Sartorius, Göttingen, Germany). Then, the filtrated extract was concentrated to dryness using a rotary evaporator at 60°C, freeze-dried, and grounded into a powder. The ETOH-ML extract was immediately stored at −80°C until further experiments.

### Phytochemical analysis of *M. longifolia* extract

The chemical analysis was conducted by using high-performance liquid chromatography (HPLC) and gas chromatography (GC). Analysis of the phenolic compounds of ETOH-ML was performed using an HPLC system (Agilent 1260 Infinity II Quat Pump, CA, USA) equipped with a photodiode array detector (PDA WR detector, CA, USA). A high-pH stable Ultisil XB-C18, 5 μm column (column dimension: length × ID = 250 mm × 4.6 mm) was used for separation. The solvent system used included solvent A (acetic acid: water, 1:99 v/v) and solvent B (methanol). The injection volume was 10 μl to detect eight compounds (gallic acid, catechin, rutin, quercetin, chlorogenic acid, caffeic acid, rosmarinic acid, and apigenin). Analyses were carried out at 30°C with a flow rate of 1.0 ml/min. The analytes were monitored using a PDA detector at 254, 280, 300, and 330 nm. The concentration of eight expected phenolic components in ETOH-ML extract was identified by comparing with retention times (Rt) and UV spectra of their respective standards (Sigma-Aldrich, Germany).

The analysis of the volatile compounds of ETOH-ML by GC was performed using an Agilent 7890 (Agilent Technologies, USA) equipped with Flame Ionization Detector (FID) and a non-polar DB-5 fused silica column (length 30 m, inner diameter 0.25 mm, and thickness of stationary phase layer equal to 0.25 μm). The initial column temperature was kept at 60°C, and then gradually increased to 260°C at a rate of 3°C/min. The injector temperature was 260°C, and nitrogen was the carrier gas at a flow rate of 1.0 ml/min. The split ratio was 10:1. The amount of components, including limonene and 1,8-cineole in ETOH-ML, was determined by injecting standard samples into GC instrument and calibration curve.

### *C. difficile* strain and culture conditions

The *C. difficile* RT001 strain that produces two toxins, TcdA and TcdB (A + B +), was obtained from the Department of Anaerobic Bacteriology at Research Institute for Gastroenterology and Liver Diseases in Tehran, Iran (Azimirad et al., 2020a). The strain was cultured on a cycloserine-cefoxitin-fructose agar (CCFA; Mast Group Ltd., Merseyside, UK) supplemented with 5% (v/v) sheep blood under anaerobic conditions (85% N_2_, 10% CO_2_, and 5% H_2_) (Anoxomat^®^ Gas Exchange System, Mart Microbiology BV) at 37°C for 48–72 h (Azimirad et al., 2022).

### Preparation of Tox-S

To obtain the toxins produced by *C. difficile* RT001 (i.e., TcdA and TcdB), Tox-S was prepared as perversely described (Azimirad et al., 2022). Briefly, *C. difficile* RT001 was grown in CCFA medium for 48 h under over-mentioned anaerobic conditions. The bacterial suspension was prepared to a 2 McFarland standard (6 × 10^8^ CFU/ml) in 0.85% sterile saline and transferred to a pre-reduced brain heart infusion (BHI) broth (Merck, Darmstadt, Germany), and incubated for 72 h at 37°C under agitation. After that, cell debris was removed by centrifugation at 4,000 × *g*, 4°C for 5 min, and the supernatant was filtered by 0.22 μm pore-sized filters, and stored at −80°C until analysis. The presence of *C. difficile* toxins A and B in the supernatant was evaluated by enzyme-linked immunosorbent assay (ELISA, Generic Assays, Germany) according to the manufacturer’s instructions. The protein concentration in Tox-S was determined using a bicinchoninic acid (BCA) protein assay kit (DNAbiotech, Tehran, Iran).

### Antimicrobial activity

#### Agar dilution assay

Antibacterial activity and minimum inhibitory concentration (MIC) of ETOH-ML were determined using agar dilution and broth microdilution assays as previously described (Azimirad et al., 2022). Different concentrations of ETOH-ML (2, 5, 10, 25, 50, 75, 100, and 200 μg/ml) were prepared by dissolving the mixture-plant extract in 10% dimethyl sulfoxide (DMSO). Moreover, the bacterial suspension was prepared to 0.5 McFarland standard (∼10^8^ CFU/ml) from pure bacterial culture on CCFA plates anaerobically at 37°C for 48 h, and further diluted to approximately 1 × 10^6^ CFU/ml using 0.85% sterile saline. The different concentrations of ETOH-ML were added in pre-reduced Brucella agar (Merck, Darmstadt, Germany). Plates were inoculated with a final concentration of approximately 1 × 10^6^ CFU/ml from the RT001 strain and inoculated at 37°C for 48 h. At the end of the incubation time, the presence or absence of bacterial growth on the plates was evaluated. The lowest concentration of the extract that inhibited the bacterial growth was determined as the MIC value. The plates containing bacterial inoculum without extract and plates without bacterial inoculation were considered as controls. The experiments were performed in triplicate and repeated three times.

#### Microdilution assay

For broth microdilution, a 96-well plate was filled with 0.5 ml sterilized pre-reduced Brucella broth (Merck, Darmstadt, Germany) and incubated with different concentrations of ETOH-ML (2–200 μg/ml) and approximately 10^5^ CFU/well of *C. difficile* RT001. The wells without extract and wells without any bacterial inoculation served as controls. Plate was then incubated under anaerobic conditions at 37°C for 48 h. The MIC value was determined for the lowest concentration of ETOH-ML, where growth inhibition was assessed by turbidity at OD_600_ nm using a microplate reader (Bio-Tek, Winooski, VT, USA). Moreover, the percentage of growth inhibition was determined by comparing the OD of treatment to control. The experiments were performed in triplicate and repeated three times.

### Cell culture and growth conditions

The Caco-2 (human colon adenocarcinoma cell line) and Vero (an African green monkey kidney cell line) cells were obtained from the Iranian Biological Resource Center (IBRC). Cells were cultured in high-glucose Dulbecco’s modified Eagle’s medium (H-DMEM; Gibco, Grand Island, NE, USA) supplemented with 10% (v/v) fetal bovine serum (FBS; Gibco, Grand Island, NE, USA), 1% (v/v) of non-essential amino acid (Gibco, Grand Island, NE, USA), and 1% (v/v) penicillin/streptomycin (Sigma-Aldrich, Darmstadt, Germany), and incubated at 37°C in a humidified CO_2_ incubator. Cultures were allowed to grow until 80% confluent.

### Cell viability assay

The effect of ETOH-ML on Caco-2 cell viability was determined using a colorimetric MTT (3-(4,5-dimethylthiazol-2-yl)-2,5-diphenyltetrazolium bromide) assay (Sigma-Aldrich, Saint Louis, MO, USA) as previously described (Azimirad et al., 2022). This technique evaluates the metabolic activity of the cells by measuring the reduction of tetrazolium salts to colored formazan products, giving the characteristic coloration to the medium. To do this, Caco-2 cells were seeded at 5 × 10^3^ cells/well in 96-well plates and treated with various concentrations (2, 5, 10, 25, 50, 75, 100, and 200 μg/ml) of ETOH-ML or Tox-S (50, 100, 250, and 500 μg/ml) and incubated at 37°C in 5% CO_2_ for 4, 8, 12, and 24 h. To determine the ability of the extract to decrease the cytotoxicity caused by Tox-S, ETOH-ML was mixed at the indicated concentrations (10 or 25 μg/ml) with Tox-S (100 μg/ml) and added to Caco-2 cells, and incubated for 4 and 24 h at 37°C in 5% CO_2_. After incubation time points, 10 μl/well of MTT was added to each well, and cells were incubated for 4 h at 37°C under a 5% CO_2_ atmosphere. After that, each well received 200 μl of DMSO for 15 min to dissolve the produced formazan crystals. Untreated monolayers were served as controls, and wells without cells were considered as blank. The plates were shaken and then incubated at 37°C for 10 min. Absorbance values were measured using a microplate reader at 570 and 630 nm (as the reference wavelength) in each well. The viability percentage was calculated as (absorbance of treated cells × 100%)/absorbance of untreated cells. The assay was carried out in triplicate.

### Cytotoxicity assay

Cytotoxic activity of ETOH-ML and Tox-S was determined using Vero cells by breakdown of the actin cytoskeleton, which leads to cell rounding, as previously described (Azimirad et al., 2022). Briefly, Vero cells were seeded in 96-well plates at 10^4^ cells/well. To estimate the cytotoxic activity, two concentrations of ETOH-ML (10 and 25 μg/ml), and Tox-S at concentration 100 μg/ml were used. To determine the ability of ETOH-ML to decrease the cytotoxicity caused by Tox-S, each concentration of the extract was mixed with Tox-S (100 μg/ml), added to the Vero cells, and incubated for 4 and 24 h at 37°C in 5% CO_2_ conditions. The cytotoxic activity was indicated using an inverted microscope (Olympus Corporation, Tokyo, Japan) at × 200 magnification. Cell images were taken, and the percentage of round cells in different treatments was determined by ImageJ software-assisted counting. The assay was carried out in triplicate.

### Total RNA extraction and RT-qPCR

Caco-2 cells were seeded into 24-well plates at a concentration of 2.5 × 10^4^ cells per well. Caco-2 cells were treated with Tox-S (100 μg/ml) alone, two concentrations of ETOH-ML (10 and 25 μg/ml) alone, and a combination of the extract (10 or 25 μg/ml) and Tox-S (100 μg/ml), and incubated for 4 and 24 h at 37°C in 5% CO_2_ conditions. Wells including untreated cells and Tox-S (100 μg/ml) without extract were used as controls. After treatments, cells were lysed for RNA extraction and gene expression analysis. All treatments were run in triplicate.

Total RNA from Caco-2 cells was extracted according to the manufacturer’s protocol of the RNeasy Mini Kit (Qiagen, Hilden, Germany). RNA purity was assessed by calculating the ratio between absorbance at 260 and 280 nm (A260/A280) using a NanoDrop spectrophotometer (Thermo Scientific, Wilmington, NC, USA). Purified RNA was reverse transcribed to cDNA using the PrimeScript™ RT Reagent Kit (Takara, Japan) according to the manufacturer’s protocol. The mRNA expression level of interleukin-1β (IL-1β), IL-8, tumor necrosis factor α (TNF-α), inducible nitric oxide synthase (iNOS), nuclear factor kappa B (NF-κB), transforming growth factor-beta (TGF-β), B-cell lymphoma 2 (Bcl-2), Bcl-2-associated X protein (Bax), and caspase-3 genes were determined using RT-qPCR assays. Gene expression assay was performed by the Rotor-Gene^®^ Q (Qiagen, Hilden, Germany) real-time PCR system using BioFACT™ 2X Real-Time PCR YBR Green Master Mix (BIOFACT, Republic of Korea). Oligonucleotide sequences used for gene expression analysis are listed in Supplementary Table 1. The β-actin housekeeping gene served as the reference gene. Relative gene expression was calculated by the 2^–ΔΔCt^ method, and the expression level was given as the fold change relative to the control sample (Livak and Schmittgen, 2001). All reactions were assessed in triplicate.

### Statistical analysis

Statistical analysis was carried out with GraphPad Prism 8.0 (GraphPad Software, San Diego, CA, USA). The data were statistically analyzed using one-way analyses of variance (ANOVAs) followed by Dunnett’s multiple range test to compare groups. The data were presented as the average of at least three independent experiments; error bars represent the standard deviations (SD). Differences were considered statistically significant when *P* < 0.05.

## Results

### Phytochemical analysis of *M. longifolia* extract

In this study, some phenolic and volatile compounds of ETOH-ML were investigated using HPLC and GC, respectively. Among eight phenolic compounds analyzed by HPLC, rosmarinic acid (1709.8 μg/ml) was the main component of ETOH-ML followed by chlorogenic acid (112.2 μg/ml) and caffeic acid (47.2 μg/ml) (Supplementary Figure 1). The amount of other analyzed compounds was undetectable. Additionally, the analysis of two volatile compounds by GC showed that 1,8-cineol (50.80 μg/ml) and limonene (10.44 μg/ml) were detected in ETOH-ML (Supplementary Figure 2).

### *M. longifolia* extract exhibits antimicrobial activity against *C. difficile* RT001

The antimicrobial activities of ETOH-ML against *C. difficile* RT001 were examined by assessing the MIC values. Based on agar dilution assay, the lowest MIC detected for ETOH-ML against *C. difficile* RT001 was observed at 25 μg/ml. ETOH-ML was further examined by broth microdilution method to determine its antimicrobial activity. Accordingly, ETOH-ML at concentration 25 μg/ml showed 100% inhibition against *C. difficile* RT001. Moreover, ETOH-ML at the concentration 10 μg/ml exhibited a percentage of inhibition of about 95%. The inhibitory activity of different concentrations of the extract against *C. difficile* RT001 are presented in Table 1.

**TABLE 1:** Inhibitory activity of *M. longifolia* extract against *C. difficile* RT001 obtained from broth microdilution method.

Extract concentration (μg/ml)	Inhibition (%)
2	10.26
5	51.44
10	95.35
25	100
50	100
75	100
100	100
200	100

### *M. longifolia* extract increases viability of Caco-2 cells stimulated by Tox-S

**FIGURE 1:**
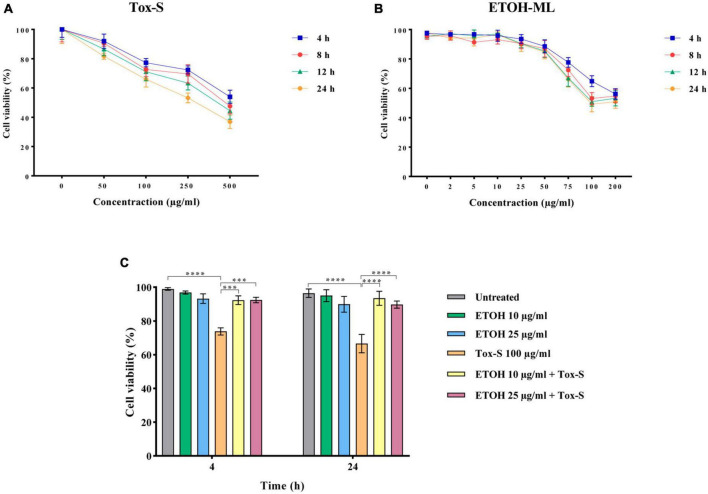
Caco-2 cell viability treated with different concentrations of **(A)** toxigenic cell-free supernatant (Tox-S) of *C. difficile* RT001 (50, 100, 250, and 500 μg/ml), and **(B)** ETOH-ML (5, 10, 25, 50, 75, 100, and 200 μg/ml), **(C)** co-treatment with ETOH-ML (10 or 25 μg/ml) and Tox-S (100 μg/ml) at 4 and 24 h. Data shown are means ± SD of three independent experiments replicated at least three times. A *P*-value of < 0.05 was considered as significant (***P < 0.01; ****P < 0.001) by unpaired student’s *t*-test and one-way ANOVA statistical analysis.

The potential of Tox-S and ETOH-ML to reduce viability was evaluated using MTT assay by incubating Caco-2 cells with different concentrations of ETOH-ML or Tox-S for 4, 8, 12, and 24 h. As presented in Figure 1A, Tox-S showed significant reduction in the number of Caco-2 cells at concentrations 100–500 μg/ml, with a dose-dependent decrease in cell viability compared to control cells. In more detail, Tox-S at concentration 100 μg/ml significantly reduced the viability of Caco-2 cells by ∼70% after 24 h (*P* < 0.01), whereas a further decrease in cell viability was observed for Tox-S at concentration 500 μg/ml (∼30% after 24 h) (*P* < 0.0001). In contrast, ETOH-ML at concentrations 5 to 25 μg/ml did not significantly decrease the viability of Caco-2 cells during incubation period, whereas higher concentrations of ETOH-ML (>50 μg/ml) reduced the cell viability after 24 h (Figure 1B). Based on these results, further experiments were carried out using 100 μg/ml Tox-S, and ETOH-ML at 10 and 25 μg/ml, due to their less cytotoxic effects on Caco-2 cells.

The co-treatment of Caco-2 cells with Tox-S (100 μg/ml) and 10 or 25 μg/ml concentration of ETOH-ML increased the viability of Tox-S-stimulated Caco-2 cells up to 90% compared with cells treated with Tox-S alone at both time points (*P* < 0.0001) (Figure 1C).

### *M. longifolia* extract exerts protective effects on Vero cells stimulated by Tox-S

**FIGURE 2:**
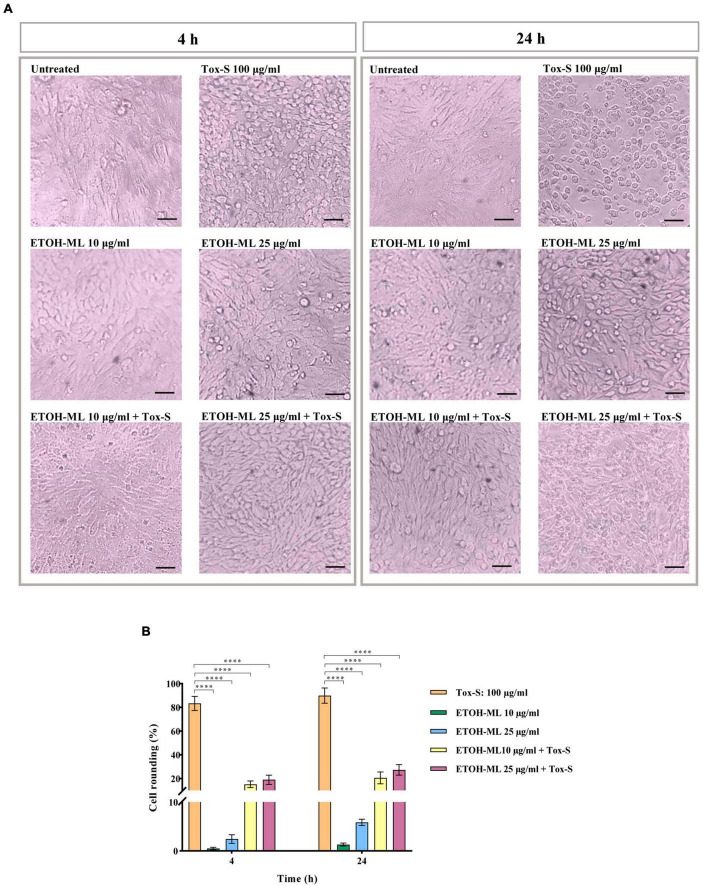
Cytopathic effect (CPE) of toxigenic cell-free supernatant (Tox-S) of *C. difficile* RT001 (100 μg/ml), two different concentrations (10 and 25 μg/ml) of ETOH-ML, and combination of Tox-S and ETOH-ML on Vero cells. **(A)** Microscopic morphology of Vero cells; and **(B)** the percentage of rounding cells after treatment with Tox-S, ETOH-ML, and co-treatment with Tox-S and ETOH-ML at 4 and 24 h. Light microscopy × 200, Scale bar = 100 μm. Data shown are means ± SD of three independent experiments. A *P*-value of < 0.05 was considered as significant (****P < 0.001) by unpaired student’s *t*-test and one-way ANOVA statistical analysis.

The effects of Tox-S of *C. difficile* RT001 and ETOH-ML on the morphology of Vero cells were investigated at both time points. Based on the morphological observation, Tox-S of *C. difficile* RT001 at concentration 100 μg/ml induced disruption of actin cytoskeleton, leading to approximately 90% cell rounding compared to untreated cells. In contrast, ETOH-ML at concentration 10 μg/ml caused no significant cell rounding, while 25 μg/ml ETOH-ML exhibited significant rounding (about 6%) after 24 h of treatment when compared to untreated cells (Figure 2A). Moreover, co-treatment of ETOH-ML and Tox-S significantly reduced the percentage of round cells induced by Tox-S compared with control cells (Figure 2A). In more detail, co-treatment of cells with Tox-S and both concentrations of ETOH-ML significantly decreased toxin-mediated cytotoxicity (>70% reduction of cell rounding) (Figure 2B). However, there was no significant difference between the protective effects of different concentrations of the extract on cell rounding.

### *M. longifolia* extract decreases mRNA expression of inflammation-related genes in Tox-S treated Caco-2 cells

**FIGURE 3:**
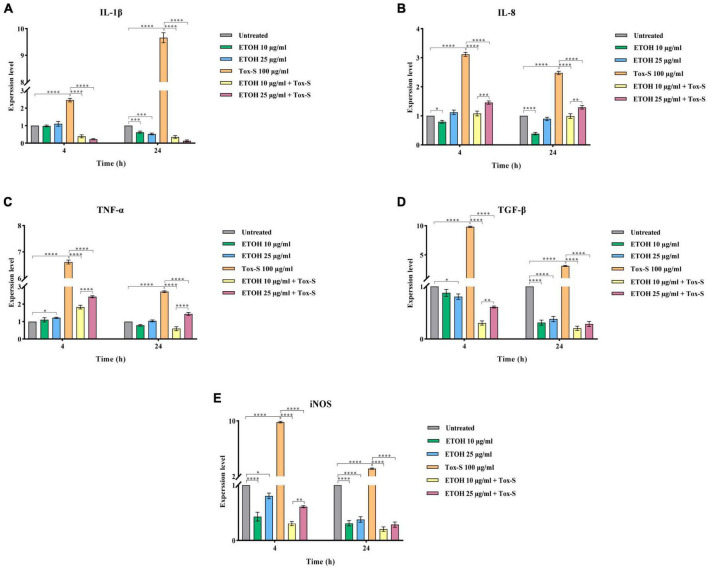
Relative expression of IL-1β **(A)**, IL-8 **(B)**, TNF-α **(C)**, TGF-β **(D)**, and iNOS **(E)** genes in Caco-2 cells upon treatment with toxigenic cell-free supernatant (Tox-S) of *C. difficile* RT001 (100 μg/ml), ETOH-ML (10 and 25 μg/ml), and co-treatment with Tox-S and ETOH-ML measured by RT-qPCR. Gene expression data was normalized to β-actin as the reference gene. Data shown are means ± SD of three independent experiments. A *P*-value of < 0.05 was considered as significant (*P < 0.01; **P < 0.05; ***P < 0.01; ****P < 0.001) by unpaired student’s *t*-test and one-way ANOVA statistical analysis.

The RT-qPCR assay was used to examine the effects of Tox-S and ETOH-ML on the expression level of inflammation-associated genes in Caco-2 cells. As shown in Figure 3, in Tox-S treated cells, the expression level of IL-1β, IL-8, TNF-α, TGF-β, and iNOS significantly increased compared to untreated cells (*P* < 0.0001). In contrast, ETOH-ML treated cells showed a significant decrease in the expression level of inflammation-related genes (except for TNF-α) at 24 h.

Co-treatment of Caco-2 cells with ETOH-ML and Tox-S significantly decreased the expression level of IL-1β, IL-8, TNF-α, TGF-β, and iNOS genes induced by Tox-S (*P* < 0.0001). Notably, ETOH-ML at concentration 10 μg/ml exerted a more significant effect on the expression level of IL-8 and TNF-α genes than concentration 25 μg/ml after 24 h (*P* < 0.01, *P* < 0.0001).

### *M. longifolia* extract decreases mRNA expression of NF-κB gene in Tox-S treated Caco-2 cells

**FIGURE 4:**
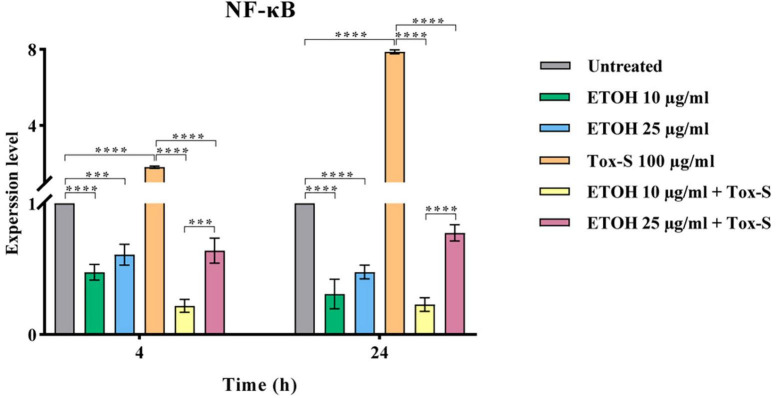
Relative expression of NF-κB gene in Caco-2 cells upon treatment with toxigenic cell-free supernatant (Tox-S) of *C. difficile* RT001 (100 μg/ml), ETOH-ML (10 and 25 μg/ml), and co-treatment with Tox-S and ETOH-ML measured by quantitative RT-qPCR. Gene expression data was normalized to β-actin as the reference gene. Data shown are means ± SD of three independent experiments. A *P*-value of < 0.05 was considered as significant (***P < 0.01; ****P < 0.001) by unpaired student’s *t*-test and one-way ANOVA statistical analysis.

To explore the anti-inflammatory potential of ETOH-ML, the expression level of NF-κB was assessed. As shown in Figure 4, Tox-S stimulated cells had an elevated expression level of NF-κB compared to control cells. Inversely, co-treatment of cells with ETOH-ML significantly reduced the gene expression of NF-κB (*P* < 0.0001). Moreover, co-treatment of Caco-2 cells with ETOH-ML and Tox-S caused a significant decrease in the expression level of NF-κB (*P* < 0.0001); however, concentration 10 μg/ml exhibited a higher inhibitory activity on the expression level of NF-κB gene than concentration 25 μg/ml after 4 and 24 h (*P* < 0.001, *P* < 0.0001).

### *M. longifolia* extract modulates mRNA expression of apoptosis-related genes in Tox-S treated Caco-2 cells

**FIGURE 5:**
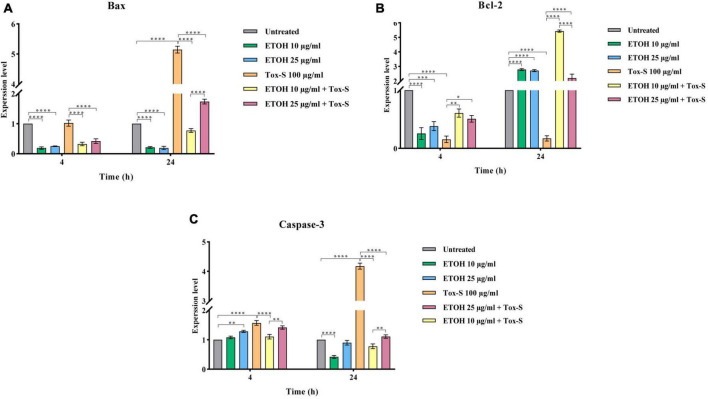
Relative expression of Bax **(A)**, Bcl-2 **(B)**, and Caspase-3 **(C)** genes in Caco-2 cells upon treatment with toxigenic cell-free supernatant (Tox-S) of *C. difficile* RT001 (100 μg/ml), ETOH-ML (10 and 25 μg/ml), and co-treatment with Tox-S and ETOH-ML measured by RT-qPCR. Gene expression data was normalized to β-actin as the reference gene. Data shown are means ± SD of three independent experiments. A *P*-value of < 0.05 was considered as significant (*P < 0.05; **P < 0.05; ***P < 0.01; ****P < 0.001) by unpaired student’s *t*-test and one-way ANOVA statistical analysis.

To determine the effect of Tox-S and ETOH-ML on apoptosis induction, the gene expression levels of Bax, Bcl-2, and caspase-3 were assessed in Caco-2 cells at the indicated time points. As shown in Figure 5, the gene expression level of Bax and caspase-3 was significantly induced by Tox-S after 24 h (*P* < 0.0001), while it was downregulated in Caco-2 cells treated with ETOH-ML at concentration 10 μg/ml (*P* < 0.0001). Notably, ETOH-ML at concentration 25 μg/ml decreased the gene expression level of Bax (*P* < 0.0001) but exerted no effect on the gene expression level of caspase-3 compared to untreated cells. In contrast, the gene expression level of Bcl-2 was significantly downregulated by Tox-S in Caco-2 cells compared to untreated cells after 24 h (*P* < 0.0001), whereas the gene expression level of Bcl-2 was significantly upregulated upon treatment with both concentration of ETOH-ML after 24 h (*P* < 0.0001).

Co-treatment of Caco-2 cells with ETOH-ML significantly decreased the gene expression level of Bax and caspase-3 induced by Tox-S during the indicated periods (*P* < 0.0001). In contrast, both concentrations of ETOH-ML significantly upregulated the gene expression level of Bcl-2 in Caco-2 cells stimulated with Tox-S after 24 h (*P* < 0.0001). Interestingly, ETOH-ML at concentration 10 μg/ml showed a higher modulatory activity on the expression level of apoptosis-related genes than concentration 25 μg/ml after 24 h (*P* < 0.01, *P* < 0.0001).

## Discussion

Previous studies have shown that toxigenic *C. difficile* strains can induce cell inflammation and apoptosis, leading to disruption of the integrity of intestinal barrier and altering intestinal morphology (Shen, 2012; Song and Kim, 2019). Based on data from our recent study, *C. difficile* RT001 has been detected as the most common *C. difficile* RT in Iranian patients with CDI (Azimirad et al., 2020a). Moreover, most of strains belonging to *C. difficile* RT001 were resistant to different antibiotics, including vancomycin, metronidazole, ciprofloxacin, moxifloxacin, and tetracycline (Baghani et al., 2020). Currently, new or alternative therapeutic approaches have been introduced for the treatment or prevention of antibiotic-resistant strains, including antibody therapy, phage therapy, and fecal microbiota transplantation (FMT) (Azimirad et al., 2020b; Raeisi et al., 2022, 2023a). Additionally, the antimicrobial efficacy of various plant extracts has been reported against antibiotic-resistant strains (Rath and Padhy, 2015; Bataineh et al., 2021; Mohanasundari et al., 2023). Several studies have been also conducted to evaluate the inhibitory effects of *M. longifolia* extracts on different bacteria (Manandhar et al., 2019; Elansary et al., 2020). Hence, the present study was conducted to investigate the antibacterial, anti-inflammatory, and anti-apoptosis activities of *M. longifolia* extract on *C. difficile* RT001 and its cytotoxicity. As shown by both agar dilution and broth microdilution assays, ETOH-ML exhibited a potent inhibitory effect on the growth of *C. difficile* RT001. The percentage of inhibitory activity of ETOH-ML against *C. difficile* RT001 showed that the extract could inhibit bacterial growth at even lower concentrations, with more than 90% inhibition at 10 μg/ml. These results are in agreement with the study performed by Elansary et al., where they demonstrated that *M. longifolia* extract exhibited significant antibacterial activity against different bacteria, including *Staphylococcus aureus*, *Bacillus cereus*, *Escherichia coli*, and *Pseudomonas aeruginosa* (Elansary et al., 2020). The presence of phenolic components and chemical properties of the extract may play a key role in the antimicrobial activity of *M. longifolia* (Farzaei et al., 2017; Bouarab-Chibane et al., 2019; Elansary et al., 2020). Based on previous studies, *M. longifolia* extract is particularly rich in flavonoids and phenolic acids, some of which exhibit antimicrobial properties, such as gallic acid, catechin, rutin, quercetin, chlorogenic acid, caffeic acid, rosmarinic acid, apigenin, 1,8-cineole, and limonene (Mkaddem et al., 2009; Snoussi et al., 2015), thus, we analyzed the presence of these components in ETOH-ML by HPLC and GC techniques. Our results indicated that ETOH-ML contained rosmarinic acid, chlorogenic acid, caffeic acid, 1,8-cineol, and limonene, some of which can penetrate microbial cells, resulting in bacterial cell disruption and death (Swamy et al., 2016). Previous studies have reported rosmarinic acid as the main phenolic component in *Mentha* species (Bahadori et al., 2018; Elansary et al., 2020). Additionally, chlorogenic acid and caffeic acid were also known as the most important components of *Mentha* species (Eftekhari et al., 2021), which are corroborated by our results. Several studies have demonstrated that these components revealed high activities against different bacterial species (Ekambaram et al., 2016; Sun et al., 2020). Moreover, other studies have also reported moderate-to-low antimicrobial activities for 1,8-cineole and limonene (Han et al., 2019; Moo et al., 2021). Interestingly, some studies have suggested that the synergistic effect of combinations of active compounds may increase the biological activity of extract against different pathogens (Huang et al., 2021). However, further research using purified components of *M. longifolia* is required to discover their precise inhibitory mode of action against *C. difficile* cells. It should be noted that there was a variation in the chemical composition of *M. longifolia* reported in our findings and previous studies, which may be due to the extraction, botanical parts of plants, geographic conditions, and harvest time (Eftekhari et al., 2021). Accordingly, some studies have reported that environment can affect phytochemical properties of extracts, however, major phenolic components identified in Iranian *M. longifolia* extracts were rosmarinic acid, chlorogenic acid, caffeic acid, which among them rosmarinic acid was detected as the predominant constituent similar to our results (Moshrefi-Araghi et al., 2021).

The cell viability results demonstrated that different concentrations of Tox-S could decrease the viability of Caco-2 cells, in agreement with a previous study (Azimirad et al., 2022). Inversely, different concentrations of the ETOH-ML had no detrimental effect on the viability of Caco-2 cells. This result was in accordance with the study performed by Yassin et al. (2020) where they reported no cytotoxicity effect for low concentrations of *M. longifolia* extract (<20 μg/ml). In our study, the use of higher concentrations of ETOH-ML (>25 μg/ml) significantly decreased the viability of Caco-2 cells up to 80%. It has been established herbal extracts at higher concentrations could lead to cell cytotoxicity (Stringaro et al., 2018; Elansary et al., 2020). Several studies have confirmed the cytotoxicity effect of *M. longifolia* extract at various concentrations (>20 μg/ml) on different cancer cell lines (Stringaro et al., 2018; Yassin et al., 2020). Previous studies have shown that high content of rosmarinic acid and other phenolic compounds in aromatic plant extracts could cause cell cytotoxicity (Luo et al., 2020; Messeha et al., 2020). Additionally, anticancer and anti-proliferative activities of chlorogenic acid and caffeic acid against different cancer cell lines have been discussed in numerous studies (Sadeghi Ekbatan et al., 2018; Zeng et al., 2021; Kimsa-Dudek et al., 2022). Since ETOH-ML at 10 and 25 μg/ml showed less cytotoxic effects on Caco-2 cells, these concentrations were used to examine the modulatory effect of the extract on cytotoxicity induced by Tox-S. Additionally, our results showed that ETOH-ML at both low and high concentrations could alleviate Tox-S-induced intestinal cell damage by increasing cell viability, indicating that the extract can decrease the cytotoxicity of Tox-S.

It is known that *C. difficile* toxins can indirectly reflect the physiological status of cells and induce cell apoptosis *in vitro* and *in vivo* (Zhang et al., 2018; Azimirad et al., 2022). Based on previous studies, *C. difficile* toxins can clearly induce cell rounding in Vero cells, which is a morphological test to evaluate cell apoptosis induced by toxins (Azimirad et al., 2022; Raeisi et al., 2023b). Our results indicated that treating Vero cells with 100 μg/ml of Tox-S could stimulate 90% cell rounding. Additionally, treatment of Vero cells with 10 μg/ml of ETOH-ML did not affect cell rounding, while 25 μg/ml of ETOH-ML showed cell rounding of about 6%. The presence of rosmarinic acid and other phenolic components in the extract can be imputable for the apoptotic activity as previously discussed (Luo et al., 2020). Interestingly, the co-treatment of ETOH-ML could strongly reduce the cell rounding of Vero cells stimulated by Tox-S. It can be suggested that ETOH-ML may enhance tight junction integrity and act as a physical barrier to cell-toxin interaction, as a result, protect the intestinal barrier against damage induced by TcdA and TcdB (Dubreuil, 2013; Shimamura et al., 2016).

As shown in previous studies, inflammation is characterized by activating intracellular signaling pathways involved in the synthesis and release of pro-inflammatory cytokines, particularly NF-κB (Chen et al., 2018). Additionally, TGF-β is a multifunctional cytokine that regulates various cellular processes such as cell growth, differentiation, and immunosuppression (Xue et al., 2020). Additionally, TGF-β can trigger apoptosis in various cell types through activating Smad-dependent pathway (Yang et al., 2021; Azimirad et al., 2022). Previous data have shown that *C. difficile* toxins can increase the expression of TGF-β1 and cytokines such as IL-8 and TNF-α, resulting in the destruction of mucosal integrity and intestinal epithelial function (Tinoco-Veras et al., 2017; Andrews et al., 2018). Based on our results, exposure of Caco-2 cells to Tox-S of *C. difficile* RT001 could upregulate the gene expression level of inflammatory cytokines, similar to other studies (Yu et al., 2017; Azimirad et al., 2022). However, co-treatment with ETOH-ML diminished the expression level of genes involved in inflammation pathways in Caco-2 cells treated by Tox-S. It has been proven that the expression of inflammatory cytokines is extensively regulated by NF-κB signaling pathway (Chen et al., 2018). Accordingly, we demonstrated that *M. longifolia* extract could modulate NF-κB activation and its downstream signaling mediators. It can be assumed that the anti-inflammatory properties of *M. longifolia* might occur through secondary metabolites such as flavonoids, alkaloids, isoprenoids, and phenolic components, which can be present in its extract at different concentrations (Mikaili et al., 2013; Farzaei et al., 2017), as previously reported by Karimian et al. (2013). In addition to cytokines, iNOS is an important target gene of NF-κB (Morgan and Liu, 2011), altered by *C. difficile* (Vedantam et al., 2012). Conversely, it has been reported that *M. longifolia* extracts could suppress iNOS mRNA expression by inactivating NF-κB, which is corroborated by our results (Karimian et al., 2013). These results provide further evidence for the anti-inflammatory effects of *M. longifolia* extract and its potential mechanism of action.

Additionally, iNOS can act as a driver of apoptosis in various cell types (Dubey et al., 2016; Nakazawa et al., 2017). The activation of apoptotic pathways is associated with changes in Bcl-2/Bax gene expression (Jan and Chaudhry, 2019). The Bax protein acts as a pro-apoptotic agent and activates a caspase cascade, resulting in apoptosis (Peña-Blanco and García-Sáez, 2018). In contrast, Bcl-2 is an anti-apoptotic protein and suppresses Bax activity (Jan and Chaudhry, 2019). According to the literature, both TcdA and TcdB from *C. difficile* strains can induce cell apoptosis *in vitro* and *in vivo* (Di Bella et al., 2016; Azimirad et al., 2022). Similarly, our results revealed that Tox-S can upregulate the gene expression of Bax and caspase-3 and downregulate the gene expression of Bcl-2, leading to the induction of apoptosis in Caco-2 cells. In contrast, ETOH-ML could modulate apoptosis in Tox-S stimulated Caco-2 cells by downregulating the gene expression level of Bax and caspase-3 and upregulating the gene expression level of Bcl-2. Similarly, our previous work showed that various plant components such as curcumin and capsaicin could exert modulatory effects on the gene expression of Bcl-2 in HT-29 cells treated by Tox-S from different *C. difficile* strains (Azimirad et al., 2022). There are a limited number of studies demonstrating the anti-apoptosis activity of plant products (Ok et al., 2017; Semwal et al., 2021). It is possible that higher concentrations of *M. longifolia* extract can induce apoptotic cell death due to higher concentrations of phenolic components in the extract, which are known for their inhibitory properties on cell growth (Anantharaju et al., 2016). However, further research is warranted to discover the pharmacokinetic profile of *M. longifolia* extract and determine its mode of action at different concentrations.

## Conclusion

**FIGURE 6:**
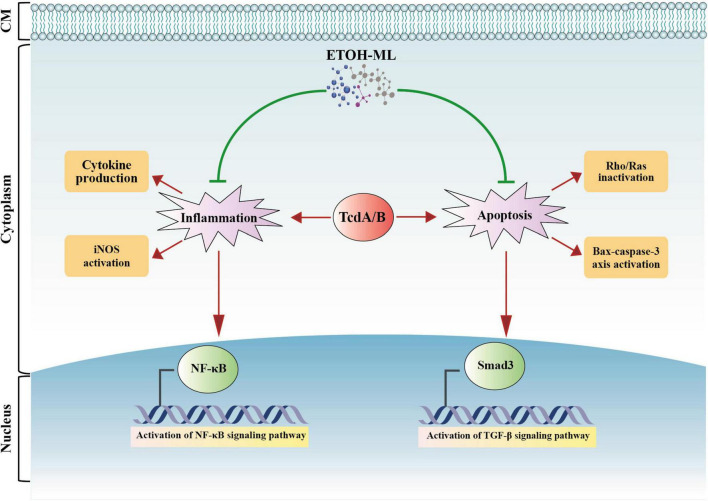
A schematic representation of biological activity of *M. longifolia* extract (ETOH-ML) on inflammation and apoptosis activated by toxins of *C. difficile*. ETOH-ML can inhibit NF-κB and TGF-β signaling pathways, which may suppress the expression of inflammatory cytokines and iNOS activity. On the other hand, ETOH-ML may inhibit activation of Rho/Ras proteins induced by toxins and downregulate Bax-caspase axis, resulting in apoptosis suppression. These outcomes can be considered as one of the potential modulatory mechanisms of ETOH-ML to suppress apoptosis and inflammatory pathways mediated by *C. difficile* toxins. Notes: red arrows indicate enhancing actions induced by toxins, whereas green lines indicate inhibitory actions induced by ETOH-ML. Bax: Bcl-2-associated X protein; CM: cell membrane; iNOS: inducible nitric oxide synthase; NF-κB: nuclear factor kappa B; TGF-β: transforming growth factor-β.

For the first time, the present study demonstrated antimicrobial activities of *M. longifolia* extract on *C. difficile* RT001, and also its modulatory effect on inflammation and apoptosis induced by Tox-S *in vitro*. Based on our findings, these modulatory effects might be mediated by suppressing the NF-κB and TGF-β signaling pathways, downregulating the Bax-caspase axis, and inhibiting the Rho/Ras inactivation pathway engaged in apoptosis regulation (Figure 6). However, one of the major limitations of the present work is the failure to measure the total composition of the extract. Further research is required to determine the biological activity of *M. longifolia* on other RTs of *C. difficile* both *in vitro* and *in vivo.* Taken together, our data can be considered as a starting point for future studies on *M. longifolia* extract as a complementary medicine to current therapies for CDI.

## Data availability statement

The original contributions presented in the study are included in the article/Supplementary material, further inquiries can be directed to the corresponding authors.

## Ethics statement

Ethical approval was not required for the studies on humans in accordance with the local legislation and institutional requirements because only commercially available established cell lines were used. Ethical approval was not required for the studies on animals in accordance with the local legislation and institutional requirements because only commercially available established cell lines were used.

## Author contributions

HR: Conceptualization, Data curation, Formal Analysis, Investigation, Methodology, Project administration, Software, Validation, Visualization, Writing – original draft, Writing – review and editing. MA: Formal Analysis, Methodology, Writing – original draft. EA: Methodology, Writing – original draft. RP: Writing – review and editing. MZ: Writing – review and editing. AY: Conceptualization, Data curation, Formal Analysis, Funding acquisition, Investigation, Project administration, Resources, Software, Software, Validation, Visualization, Writing – review and editing.
