# Supplementary material for: Pleiotropic effects of Mentha longifolia L. extract on the regulation of genes involved in inflammation and apoptosis induced by Clostridioides difficile ribotype 001
Source: Front Microbiol. 2023 Oct 27;14:1273094. doi: 10.3389/fmicb.2023.1273094 (PMC10641701; doi:10.3389/fmicb.2023.1273094)
Supplement: Supplementary file 1 [file Data_Sheet_1.PDF]

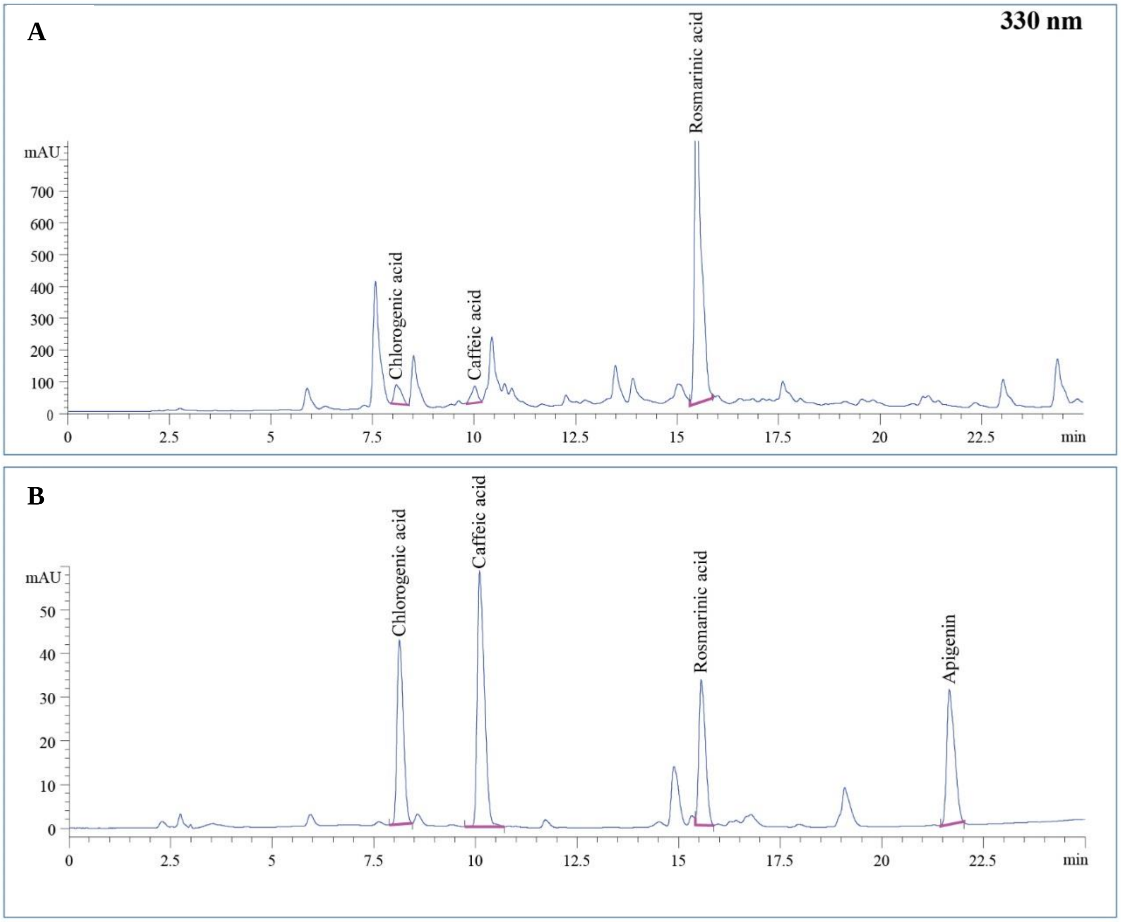

Supplementary Figure 1. Typical HPLC chromatogram of *M. longifolia* extract **(**ETOH-ML). Key components were identified based on the identical retention times as those of the standards for chlorogenic acid, caffeic acid, rosmarinic acid, and apigenin. The chromatograms of (**A**) ETOH-ML, and (**B**) standards in 330 nm.

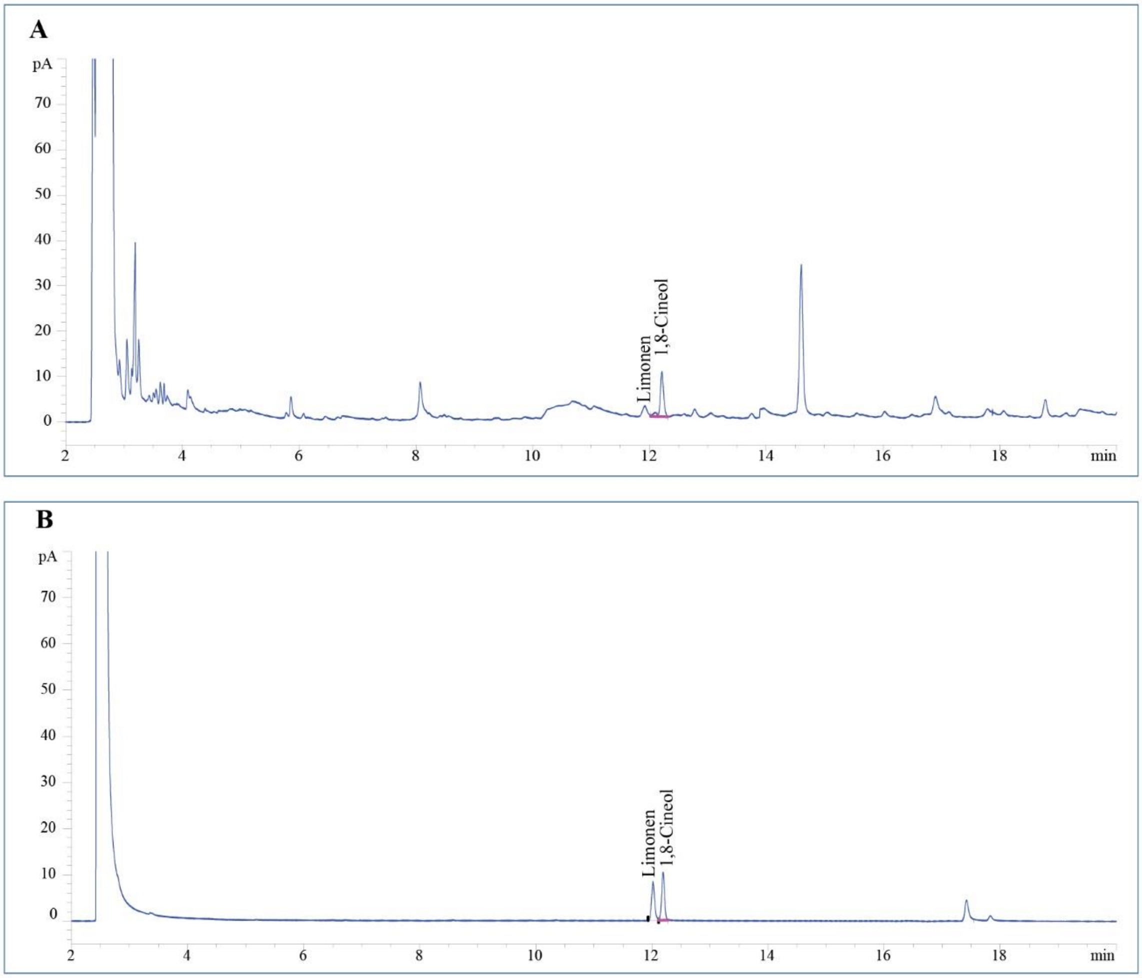

Supplementary Figure 2. Typical GC chromatogram of *M. longifolia* extract (ETOH-ML). Key components were identified based on the identical retention times as those of the standards for limonene and 1,8-cineol. The chromatograms of (**A**) ETOH-ML, and (**B**) standards.

Supplementary Table 1 Oligonucleotide sequences used in this study.

Target gene	Oligonucleotide sequence (5′-3′)	T_m °C	Reference
IL-1β	F: AACAACTACTCAGAAACACAAG<br>R: CAGAACTCAGGAATGGA	59	[1]
IL-8	F: AGCACTCCTTGGCAAAACTG<br>R: CGGAAGGAACCATCTCACTG	60	[2]
TNF-α	F: AGCCCATGTTGTAGCAAACC<br>R: TGAGGTACAGGCCCTCTGAT	56	[3]
NF-κB	F: GTATTTCAACCACAGATGGCACT<br>R: AACCTTTGCTGGTCCCACAT	55	[4]
TGF-β	F: CAATTCCTGGCGATACCTCAG<br>R: GCACAACTCCGGTGACATCAA	56	[4]
iNOS	F: AGACTGGATTTGGCTGGTCCCTCC<br>R: AGAACTGAGGGTACATGCTGGAGCC	56	[5]
Bax	F: CCTGTGCACCAAGGTGCCGGAACT<br>R: CCACCCTGGTCTTGGATCCAGCCC	57	[6]
Bcl-2	F: GAGCTGGTGGTTGACTTTCTC<br>R: TCCATCTCCGATTCAGTCCCT	55	[6]
Caspase-3	F: ACATGGCGTGTCATAAAATACC<br>R: CACAAAGCGACTGGATGAAC	57	[7]
β-actin	F: ATGTGGCCGAGGACTTTGATT<br>R: AGTGGGGTGGCTTTTAGGATG	59	[8]

References

1. Raftar, S. K. A., Ashrafian, F., Abdollahiyan, S., Yadegar, A., Moradi, H. R., Masoumi, M., Vaziri, F., Moshiri, A., Siadat, S. D., Zali, M. R. (2022). The anti-inflammatory effects of Akkermansia muciniphila and its derivates in HFD/CCL4-induced murine model of liver injury. *Sci. Rep*. 12(1), 2453. https://doi.org/10.1038/s41598-022-06414-1
2. Zhao, S., Guo, J., Zhao, Y., Fei, C., Zheng, Q., Li, X., Chang, C. (2016). Chidamide, a novel histone deacetylase inhibitor, inhibits the viability of MDS and AML cells by suppressing JAK2/STAT3 signaling. *Am. J. Transl. Res*. 8(7), 3169-3178.

3. Jin, Y., Lu, X., Wang, M., Zhao, X., Xue, L. (2019). X-linked inhibitor of apoptosis protein accelerates migration by inducing epithelial–mesenchymal transition through TGF-β signaling pathway in esophageal cancer cells. *Cell Biosci*. 9(1):76. https://doi.org/10.1186/s13578-019-0338-3
4. Kouser, L., Paudyal, B., Kaur, A., Stenbeck, G., Jones, L. A., Abozaid, S. M., Stover, C. M., Flahaut, E., Sim, R. B., Kishore, U. (2018). Human Properdin Opsonizes Nanoparticles and Triggers a Potent Pro-inflammatory Response by Macrophages without Involving Complement Activation. *Front. Immunol.*https://doi.org/10.3389/fimmu.2018.00131
5. Ulbrich, S. E., Rehfeld, S., Bauersachs, S., Wolf, E., Rottmayer, R., Hiendleder, S., Vermehren, M., Sinowatz, F., Meyer, H. H. D., Einspanier, R. (2006). Region-specific expression of nitric oxide synthases in the bovine oviduct during the oestrous cycle and *in vitro*. *J. Endocrinol*. 188(2), 205-213. https://doi.org/10.1677/joe.1.06526
6. Aghdaei, H. A., Kadijani, A. A., Sorrentino, D., Mirzaei, A., Shahrokh, S., Balaii, H., Geraci, M., Zali, M. R. (2018). An increased Bax/Bcl-2 ratio in circulating inflammatory cells predicts primary response to infliximab in inflammatory bowel disease patients. *United Eur. Gastroenterol. J*. 6(7):1074-81. https://doi.org/10.1177/2050640618774637
7. Karimi Ardestani, S., Tafvizi, F., Tajabadi Ebrahimi, M. (2019). Heat-killed probiotic bacteria induce apoptosis of HT-29 human colon adenocarcinoma cell line via the regulation of Bax/Bcl2 and caspases pathway. *Hum. Exp. Toxicol*. 38(9):1069-81. https://doi.org/10.1177/0960327119851255
8. Ofinran, O., Bose, U., Hay, D., Abdul, S., Tufatelli, C., Khan, R. (2016). Selection of suitable reference genes for gene expression studies in normal human ovarian tissues, borderline ovarian tumours and ovarian cancer. *Mol. Med. Rep*. 14(6), 5725-5731. https://doi.org/10.3892/mmr.2016.5933
